# The Outcome of Scaphoid Fracture Nonunion Managed by 1,2 Intercompartmental Supraretinacular Artery (1,2 ICSRA) Vascularized Bone Graft

**DOI:** 10.7759/cureus.47489

**Published:** 2023-10-22

**Authors:** Md Mohiuddin, Bipul K Das, Raquib M Manzur, Jahangir Alam, Syed E Shaude

**Affiliations:** 1 Department of Hand and Microsurgery, National Institute of Traumatology and Orthopaedic Rehabilitation (NITOR), Dhaka, BGD; 2 Department of Orthopaedic Surgery, National Institute of Traumatology and Orthopaedic Rehabilitation (NITOR), Dhaka, BGD; 3 Department of Research and Development, International Network of Doctors Journal, Dhaka, BGD

**Keywords:** avascular necrosis, treatment, outcome, vascularized bone graft, avn, scaphoid non-union

## Abstract

Background: Scaphoid fracture is most often missed and mismanaged leading to scaphoid nonunion with or without avascular necrosis. When avascular necrosis of the proximal pole is confirmed with intraoperative evaluation, conventional bone graft is not enough. The treatment modalities are evolving day by day. The current trend is vascular bone grafting, which has shown good outcomes in terms of union and wrist function.

Methods: Fifty patients with nonunion fracture of the scaphoid were treated with vascularized pedicle bone graft from the dorsum of the distal radius using the 1st and 2nd intercompartmental supraretinacular artery, from 2014 to 2022. Preoperative and postoperative clinical evaluation included pain, range of motion, grip strength, and satisfaction. The average follow-up period was 12 months.

Results: Among 18 patients, 14 were clinically improved after a mean follow-up period of eight weeks. Thirteen patients reported the absence of any discomfort, three patients reported slight discomfort after hard work, and two patients reported pain with light work. The wrist range of motion improved significantly, and the hand grip strength also improved. According to the modified Mayo wrist scoring chart, clinical results were rated as excellent in 24 cases, good in 19 cases, and poor in four cases.

Conclusion: 1,2 intercompartmental supraretinacular artery (1,2 ICSRA) is superficial to the extensor retinaculum and is a proper pedicle of vascularized bone graft due to the ease of visibility and dissection. The functional results and union rates were satisfactory in our study.

## Introduction

Scaphoid fractures represent less than 2% of all fractures [1], but they are the most common fractures among the carpal bones [2]. Up to 40% of acute scaphoid fractures may be missed at initial radiographs [3]. Thereby, a significant portion (5-15%) of all acute fractures may undergo nonunion and subsequently result in avascular necrosis (AVN) [4]. Failure of initial diagnosis, delay in treatment, lack of initial immobilization, proximal location, displacement of fractures, and peculiarity of vascular anatomy may contribute to scaphoid nonunion and may result in AVN of scaphoid, wrist arthritis, and carpal instability, if not treated properly [2-4].

According to Lichtman [5], scaphoid nonunion can be classified as type I-V, where type I is simple, type II is unstable, and others are degenerative. There are many treatment options for scaphoid nonunion, such as prolonged immobilization, electromagnetic therapy, salvage surgery, nonvascularized bone graft, and vascularized bone graft, which may be pedicled or free [5]. At present, several authors in different studies found excellent results with pedicle vascularized bone [6-11]. Vascularized bone grafts are indicated in small proximal pole nonunion and long-standing nonunion of the scaphoid waist, AVN [5], or failed nonvascularized grafts [6]. Primary vascularized bone graft in scaphoid nonunion is justified by some authors as it removes the fear of long-standing immobilization and less soft tissue damage that could occur from previous failed surgeries [5]. The rationale for using this are short period of immobilization and a higher union rate, as living bone tissue heals faster without creeping substitution of necrotic bone tissue and adequate blood supply may allow revascularization in avascular bones [12]; furthermore, it also gives strength, toughness, and greater elasticity than nonvascularized graft [13]. Besides these, vascularized bone graft also allows dorsal intercalated segment instability (DISI) malalignment to be corrected, which permits early motion exercises, wrist extension in particular [5]. It was reported that 5° of scaphoid angulation results in 24% of wrist extension and total loss of extension results from 15° of dorsal angulation [14]. Therefore, the aim of the study was to evaluate the outcome of 1,2 intercompartmental supraretinacular artery (1,2 ICSRA) as primary vascularized grafts for the treatment of scaphoid nonunion. Lichtman types of scaphoid nonunion are as follows: (i) type I: simple and no displacement; (ii) type II: unstable and displacement >1 mm/scapholunate angle >70°; (iii) type III: early arthritic and radioscaphoid arthritis; (iv) type IV: scaphoid nonunion advanced collapse and radioscaphoid and midcarpal arthritis; (v) type V: scaphoid nonunion advanced collapse plus arthritis throughout wrist.

## Materials and methods

The study was a prospective and multicenter study done at the National Institute of Traumatology and Orthopaedic Rehabilitation (NITOR) and one private hospital outside NITOR, Dhaka, Bangladesh from 2015 to 2022. Fifty participants who had scaphoid nonunion and met the selection criteria were selected. Lichtman type I and type II of scaphoid nonunion were included in the study. Patients with scaphoid nonunion with degenerative changes were excluded from the study. Preoperatively, all of them were evaluated both clinically and radiologically. Nonunion was defined as a fracture gap with a sclerotic zone on X-ray. Surgery was done following the technique described by Zaidemberg et al. in 1991 [7]. Postoperatively, patients were followed up in the second week, third month, sixth month, and 12th month. All of them were evaluated clinically by observing pain level, satisfaction, grip strength, and range of movement of the wrist and compared with the opposite healthy site. Radiologically, the disappearance of the fracture line was considered a union at the final follow-up. Finally, all patients were followed up by a modified Mayo wrist scoring system. Data were collected in a pre-set questionnaire, tabulated, and analyzed by SPSS version 24 (IBM Corp., Armonk, NY). An unpaired Student's t-test was done to measure the significance of the results. A p-value of 0.05 was considered statistically significant. Ethical approval was obtained from the Institutional Review Board (IRB) of the National Institute of Traumatology and Orthopaedic Rehabilitation (NITOR), Dhaka, Bangladesh (NITOR/IRB/2020/1025).

Clinical evaluation

Patients with scaphoid nonunion usually present with a history of previous trauma, which may be a fall on the outstretched hand or hyperextension injury to the wrist with initial inadequate or no treatment. These patients complain of decreased wrist motion, weakness, inability to perform pushups, or radial site wrist pain. If the scaphoid nonunion remains untreated, it may lead to AVN of the scaphoid, which may result in carpal collapse, humpback deformity, or dorsal intercalated segmental instability in long-standing cases [4].

Imaging

Up to 20% of acute scaphoid fractures can be missed in initial X-rays [2]. Despite this, X-rays of the wrist (posteroanterior, lateral, and oblique), scaphoid (posteroanterior view of the wrist with ulnar deviation), and clenched fist (anteroposterior view) are the standard X-ray views to diagnose scaphoid fractures and nonunion [4]. Union was defined in this study as the absence of a fracture gap with the restoration of the trabecular network [15], and nonunion was defined in this study as fracture without healing and fracture gap with sclerotic zone on X-ray [16]. The severity of complications was determined by intrascaphoid (IS) and scapholunate (SL) angle and compared both pre and postoperatively [12].

MRI is very useful to diagnose nonunion and AVN. Low signal intensity on T1-weighted images and high signal intensity on T2-weighted images on MRI are suggestive of AVN. However, the absence of punctate bleeding intraoperatively confirms AVN [12]. CT scan is also useful, but it is mostly useful in the diagnosis of fractures in the initial stages and also in monitoring fracture healing in the postoperative period.

Surgical technique

**Figure 1:**
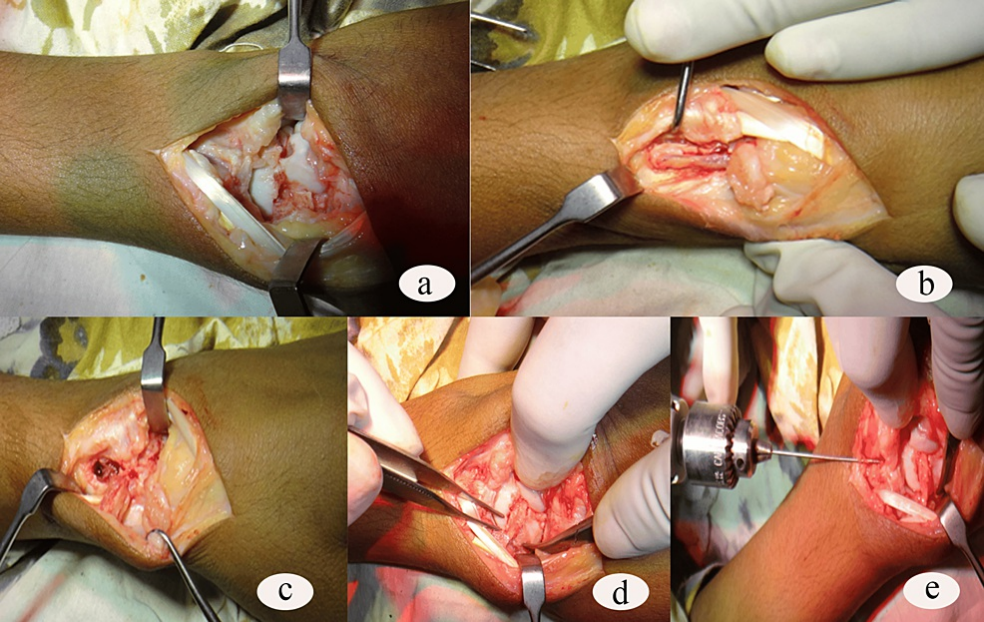
(a) Preparation of fracture site. (b & c) Harvesting of vascularized graft. (d) Inset of the graft to the fracture site. (e) Insertion of guidewire.

This technique of vascularized bone graft was first described by Zaidemberg et al. in 1991 [7]. With the patient under appropriate anesthesia and after elevating the extremity, a tourniquet was inflated. A curvilinear dorsoradial incision was made. After raising the subcutaneous tissues from the retinaculum, the 1,2 ICSRA was visualized on the surface of the extensor retinaculum. This artery was then inspected using loupe magnification. The artery and venae comitantes were dissected gently toward their distal anastomosis. After identification and protection of the sensory branches of the radial nerve, an interval was developed between the first and second dorsal compartments that were opened at the graft elevation site. Before the graft was elevated, the scaphoid nonunion site was prepared. A trough for a dorsal inlay graft was prepared. A graft of adequate length was raised containing the vessels to fill the defect of the scaphoid. The center of the graft was 1.5 cm proximal to the radiocarpal joint to include the vessels. The tourniquet was briefly released to check the bleeding from the vascularized bone graft. Then the tourniquet was reinflated and the graft was placed on the nonunion site as a dorsal inlay graft. In addition to this, some cancellous bone grafts were taken from the radius to fill the remaining defect of the scaphoid. Then a guidewire was passed across the graft and fracture site. Then the fracture site was fixed by a 2.5 mm Herbert screw of adequate length (20-22 mm) from proximal to distal direction. Fixation was confirmed by the image intensifier preoperatively. Then the wound was closed in layers keeping the drain in place. The drain was removed on the second postoperative day. Stitches were removed on the 14th postoperative day. Scaphoid plaster was applied, which was removed in the sixth week. A full range of movement exercises were allowed (Figure 1).

Postoperative evaluation

Radiological

Posteroanterior and lateral views were taken postoperatively. The disappearance of the fracture line and trabecular realignment was considered a union of the fracture.

Functional

A modified Mayo wrist scoring system was used to evaluate functional outcomes postoperatively [12]. The Mayo wrist score consists of pain, range of motion, and grip strength. With the addition of the satisfaction score, the scoring system was modified, which was used in this study. Range of motion consists of flexion, extension, radial deviation, and ulnar deviation. This range of motion was compared to a contralateral healthy site. Grip strength was measured with a dynamometer and reported as a percentage of the maximal strength of the opposite site.

## Results

**Figure 2:**
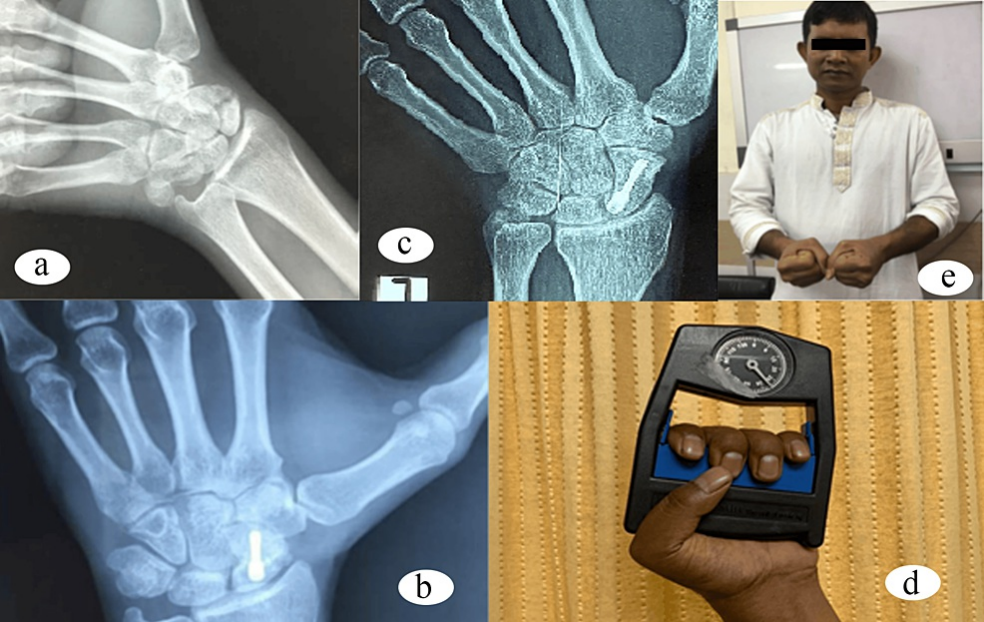
(a) Preoperative image showing fracture at the waist of scaphoid. (b) Follow-up image three months following surgery. (c) Image taken six months after surgery. (d) Measuring grip strength at final follow-up. (e) Range of movement of the wrist six months after surgery.

In this study, we operated scaphoid fracture nonunion of 50 respondents. Of them, 45 were male, and most of them (38%) were between 40 and 49 years of age. The right hand was predominant (60%). The mean delay of surgery from the time of injury was 8.50 ± 9.37 (mean ± SD) months, where the majority of the respondents were operated on in less than six months (40%) following injury. Thirty (60%) respondents received no previous treatment, whereas 18 of them were managed in the form of cast application previously. All the respondents were evaluated with X-ray and MRI preoperatively. Waist fracture was found in 42 (84%) respondents, and the rest of them had proximal pole fracture (16%). The majority of them were classified as Lichtman type I (60%). Twenty (40%) of them had developed AVN preoperatively. All of the respondents were treated with 1,2 ICSRA as primary vascularized bone graft and fixed with Herbert screw. The mean time of union was 6.74 ± 1.71 (mean ± SD) weeks, where 40 (80%) were united in six weeks. The final range of movements (flexion, extension, radial deviation, and ulnar deviation) was compared with the opposite healthy site where the p-value was 0.002 (<0.05), which was statistically significant. Finally, the respondents were evaluated by the modified Mayo wrist score. According to these scores, 24 (48%) respondents had excellent results, 19 (38%) had good results, and four (8%) of them had poor results (Figure 2).

Table 1 shows the distribution of pre and postoperative variables of the study population. Here, the right site was 30 (60%) and the left site was 20 (40%). According to the delay of treatment, <6 were 80%, 6-18 were 8%, 19-30 were 6%, 31-42 were 1%, 43-54 were 4%, and >54 were 1%. Based on fracture position, 42 (84%) were waist fractures and eight (16%) were proximal pole fractures. According to preoperative AVN, 20 (40%) were positive and 30 (60%) were negative.

**Table 1:** Distribution of pre and postoperative variables.

Variables		n = 50	Percentage (%)
Site	Right	30	60
	Left	20	40
Delay of treatment (in months)	<6	40	80
	6-18	3	8
	19-30	3	6
	31-42	1	1
	43-54	1	4
	>54	1	1
Previous treatment	No previous treatment	30	60
	Nonoperative treatment	20	40
Lichtman type	Type 1	30	60
	Type 2	20	40
Fracture position	Waist	42	84
	Proximal pole	8	16
Preoperative avascular necrosis	Yes	20	40
	No	30	60
Union time (in weeks)	<6	40	80
	6-12	8	16
	>12	2	4

Table 2 shows the final movement of the wrist. Here, according to the injured site, flexion, extension, radial deviation, and ulnar deviation were 85°, 60°, 15°, and 40°, respectively. According to the healthy site, flexion, extension, radial deviation, and ulnar deviation were 90°, 70°, 20°, and 50°, respectively (p-value = 0.002).

**Table 2:** Final movement of the wrist (compared to the healthy site).

	Injured site	Healthy site	P-value
Flexion	85°	90°	
Extension	60°	70°	0.002
Radial deviation	15°	20°	
Ulnar deviation	40°	50°	

**Figure 3:**
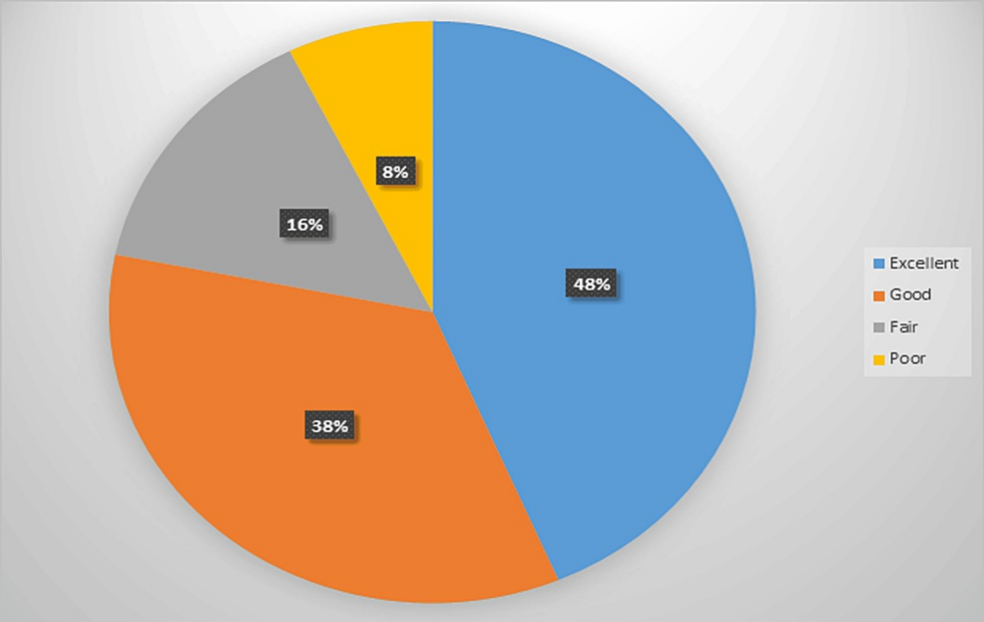
Distribution of results according to the modified Mayo wrist score.

Figure 3 shows the distribution of results according to the modified Mayo wrist score. Here, 48% had excellent results, 38% had good results, 16% had fair results, and 8% had poor results.

## Discussion

Scaphoid is the most common carpal bone to be fractured. The fracture is often missed in the initial presentation, which may lead to nonunion and subsequently AVN. Conventional Russe [17] inlay bone graft achieved union in 70-90% of cases [18], but a recent meta-analysis by Merrell et al. [19] showed that this non-vascularized bone graft is only 47% effective in achieving union if there is AVN, whereas vascularized bone graft achieved union in 88% of cases in the presence of AVN. Green [18] found 92% union in proximal pole fractures with good vascularity, which were treated with conventional graft, but none of his five patients with proximal pole AVN achieved union. In conclusion, he suggested vascularized bone graft in scaphoid nonunion with AVN cases. Though Smith and Cooney [3] suggested an algorithm for the treatment of scaphoid nonunion, in this study, we used a primary vascularized bone graft to avoid long-term immobilization and soft tissue damage from the second surgery. Furthermore, vascularized bone graft replaces the creeping substitutes, provides a bony bridge, and acts as a source of living osteogenic tissues, thereby allowing revascularization of the avascular part of the bone.

Several authors used different methods and different donor sites for vascularized bone grafts. Chacha [10] and Kawai and Yamamoto [11] used bone graft and vascular pedicle from pronator quadratus, Mathoulin et al. [8] harvested vascularized bone graft from index metacarpal, Kuhlmann et al. [20] took the graft based on the volar carpal artery, whereas Mathoulin et al. [9] used a palmar carpal artery pedicled bone graft. Each of them has specific advantages and disadvantages. Zaidemberg et al. [7] used 1,2 ICSRA as the pedicle of the bone graft due to its easy identification and sufficient arc of rotation. In their study, all of the 11 cases united in 6.2 weeks with good functional results. They recommended its use in long-standing cases of scaphoid nonunion. Uerpairojkit et al. [5] also used a vascularized bone graft from the dorsoradial aspect of the distal radius. They found radiographic union in 6.5 weeks, where five cases of AVN united at four weeks. They found no donor site morbidity. They concluded that a short time of healing permitted early mobilization and resulted in a good range of motion. They believed the use of vascularized bone graft in nonunion as a primary graft to avoid a second surgery.

In this study, the mean time of union was 6.74 weeks. According to the modified Mayo wrist score, 86% of the respondents had excellent and good results in the present study. Four of them had poor results in terms of pain, grip strength, and range of motion. This might be due to the location of the fracture in the proximal pole with late presentation and inadequate postoperative rehabilitation. This study used the 1,2 ICSRA as pedicle vascularized bone graft only, so a comparative study using free medial femoral condyle graft could be done for further study.

## Conclusions

Due to superficial location and ease of dissection, it may be concluded that vascularized bone graft using 1,2 ICSRA was useful in treating scaphoid fracture nonunion and AVN. Special surgical experience is required for this surgical technique. This study determined that the probability of a successful outcome is different and depends on the most effective surgical technique and careful patient and fracture selection.
